# Outcomes in older blunt chest wall trauma patients: a retrospective study

**DOI:** 10.1186/cc11060

**Published:** 2012-03-20

**Authors:** C Battle, H Hutchings, PA Evans

**Affiliations:** 1 Swansea University, Swansea, UK

## Introduction

Blunt chest wall trauma accounts for over 15% of all trauma admissions to emergency departments in the UK and has high morbidity and mortality rates [1]. Reported risk factors for morbidity and mortality in blunt chest trauma patients include patient age, pre-existing disease and three or more rib fractures [2]. No guidelines exist for management of this patient group unless the patient has severe immediate life-threatening injuries. The aim of this study was to investigate whether blunt chest wall trauma patients aged 65 years or more have higher rates of mortality, morbidity (respiratory complications), ICU admissions and hospital length of stay (HLOS) than patients aged less than 65 years.

## Methods

A retrospective study was completed in which the notes of 1,056 blunt chest wall trauma patients who presented in 2010 to the emergency department of a large regional trauma centre in Wales were examined. A total of 94 out of the 1,056 (9%) patients were admitted to hospital in 2010 with blunt chest wall trauma. Data were recorded for each of the admitted patients including patient age, severity of injury, morbidity, mortality, ICU admission and HLOS. Patients were grouped according to age; group one included all blunt chest wall trauma patients aged 65 years or more and group two included all patients aged less than 65 years. Pearson's chi-square analyses were performed to determine whether any differences existed between the two groups and significance set at *P *< 0.05.

## Results

There was no significant difference in severity of injury between the groups. The mortality rate and HLOS in the patients aged 65 years or more were significantly higher (*P *< 0.05) than in the younger patient group. There were no significant differences between the morbidity rates and number of ICU admissions.

## Conclusion

Blunt chest wall trauma patients have a significantly higher rate of mortality and hospital length of stay if aged 65 years or more when compared to those patients aged less than 65 years. Older blunt chest wall trauma patients should be considered for a higher level of care on admission to hospital from the emergency department.
